# Incidental finding of COVID-19 infection amongst staff at a primary care facility in Ghana

**DOI:** 10.4102/phcfm.v12i1.2669

**Published:** 2020-10-09

**Authors:** Priscilla Vandyck-Sey, Gordon Amoh, Akye Essuman, Henry Lawson

**Affiliations:** 1 Faculty of Family Medicine, Korle Bu Polyclinic and Family Medicine Centre, Korle Bu Teaching Hospital, Accra, Ghana; 2 Family Medicine Unit, Department of Community Health, University of Ghana Medical School, Accra, Ghana

**Keywords:** COVID-19 infection, healthcare worker, staff, Ghana, incidental finding, modified PPE

## Abstract

The COVID-19 pandemic has affected nearly every country worldwide and all African countries. The issue of healthcare workers (HCWs) contracting the disease is a growing concern in Ghana, because of the risk of spreading infections amongst themselves and to vulnerable patients in their care. This article illustrates how 14 staff at the Korle Bu Polyclinic/Family Medicine Department were incidentally found to be Covid-19 positive with most of them being asymptomatic. This observation led to a modification of the personal protective equipment (PPE) used by clinical staff when attending to patients. Furthermore, this finding suggests that a different criteria or guideline may be needed for testing of HCWs during a pandemic where a significant proportion of infected people are asymptomatic. We conclude that in the primary care setting HCWs must be ready to see all the following cases safely: routine patients, asymptomatic COVID-19 patients and suspected COVID-19 patients.

## Background

Ghana’s first two cases of COVID-19 infections were reported on 12 March 2020, the current situation stands at 37 812 cases, with 34 313 recoveries and 191 deaths as at 31 July 2020.^1^ Data from the Ghana Health Service (GHS) suggest that there is active community transmission and the first asymptomatic cases were reported on 15 April 2020.^2^ Infections in healthcare workers (HCWs) are a growing concern and a number of reports have been made across the country. The Ghana News Agency reported in June 2020 that about 110 HCWs had been infected^3^ whilst *The Ghana Report*, published on 04 July 2020, stated that over 150 HCWs had been infected with six deaths including four doctors.^4^ In addition, GHS is said to have confirmed that over 2000 HCWs have been infected with COVID-19 after the first two cases in Ghana was reported by CNN edition.^5^

## Purpose

The Korle Bu Polyclinic/Family Medicine Department (KPFMD) is the Family Medicine training centre and provider of primary care at Korle Bu Teaching Hospital (KBTH). This article will share how some staff at the department were incidentally found to be Covid-19 positive and how the department handled the situation.

## Path to discovery and actions

On 28 March 2020, a 28-year-old female patient (A.B.) with no known medical conditions, visited the KPFMD around 5:00 pm. On 03 April 2020, the KPFMD discovered that A.B. was a primary contact of a HCW in another department in KBTH, who had tested positive for COVID-19 disease. A retrospective review of A.B.’s clinical notes revealed that she had presented at our Out-Patient Department (OPD) with symptoms of fever (37.9 °C), general malaise and chills. The patient A.B. neither had an international travel history nor contact with a person who had recently travelled. She had no headache, cough, sore throat, running nose or any other symptoms suggestive of Covid-19 disease and hence, was treated as a routine case. After a normal physical examination, testing negative for malaria and a normal urinalysis, she was given a clinical diagnosis of ‘Fever – unspecified’ and was managed on outpatient basis with oral paracetamol and vitamin C. She visited only the OPD and laboratory at presentation and was at the KPFMD for approximately 3 h and 30 min (5:00 pm – 8:30 pm).

At the time we made the discovery that A.B. was a primary contact to a confirmed case, her sample had already been taken for COVID-19 testing by the Public health team of KBTH. However, her results were not yet ready, hence, possible contacts of A.B. in our department could not be screened then. Moreover, if outcome of her test was negative, no staff screening would be necessary and if positive, staff will then need to be screened. Four days later (07 April 2020), A.B.’s status changed to a confirmed case of COVID-19 disease. It had then been 10 days since A.B. presented and some staff could not remember whether they had come into direct contact with A.B or not. Subsequently, 43 clinical and non-clinical staff on duty in the afternoon and night on the day A.B. presented were screened on 09 April 2020. This was day 12 after the ‘exposure’. Nasopharyngeal washings using 5 mL of normal saline were used to collect samples for screening. Screening was organised by the KBTH COVID-19 taskforce team under the Public Health Unit and the samples were run at the National Public Health Reference Laboratory. Results were ready after 7 days.

### Ethical consideration

Permission to use secondary data was sought from hospital authorities.

## Results

Because of the outcome of results of these 43 staff (see Table 1), a number of actions were taken:

**TABLE 1:** Positivity rate of staff screening.

Staff description	Total screened for COVID-19	Number Negative	Number positive	Number with inadequate sample/need to repeat	Positivity rate (%)	Unit(s) with staff testing positive	Number positive in units
Staff on afternoon and evening duty on 28 March 2020	43	27	6	10	14	OPD	3
						Male ward	1
						Records	1
						Bank	1
						Security	1
All other Staff screening-1	236	231	5	Nil	2.1	Hospitality	2
						Pharmacy	1
						Laboratory	1
						Female ward	1
All other Staff screening-2 (Mop-up)	39	36	3	Nil	7.7	Hospitality	1
						Bank	1
						ICT	1
**Overall total**	**318**	**294**	**14**	**10**	**4.4**	**10 units**	**14**

OPD, Out-Patient Department.

1. All other staff in the KPFMD were screened for COVID-19 disease because it was apparent that there were staff who were asymptomatic carriers in some units (Tables 1 and 2).
2. On 17 April 2020, 20 days (20) after A.B.’s visit, the KPFMD was closed down for cleaning and disinfection whilst awaiting COVID-19 test results.
3. Staff who tested positive for the COVID-19 virus were admitted to designated national COVID-19 treatment centres for further management.
4. The KPFMD reopened on 29 April 2020 with staff who tested negative.
5. A second (mop-up) screening for all staff who missed the initial screening-1 was also conducted on 07 May 2020 (Table 1).
6. We reduced our work shift from three (8 am – 2 pm, 2 pm – 8 pm and 8 pm – 8 am) to two 12-h shifts (8 am – 8 pm and 8 pm – 8 am) per day.
7. All clinical staff were instructed to wear goggles or face shields in addition to medical/surgical masks and reusable surgical gowns with disposable rubber aprons whilst attending to routine cases and suspected cases of COVID-19. Hazmat suits, N-95 masks with head covers, aprons and shoe covers were to be worn when reviewing detained suspected cases.

## Discussion

The patient record of A.B. showed that staff who had proven evidence of contact such as the nurse who checked her vital signs, the doctor she consulted and the laboratory technician who took her blood sample all tested negative. Some staff who tested positive were not directly involved in A.B.’s care but were screened because they were on duty on the said date. The positivity rate amongst the initially ‘exposed’ staff, (14%) was about 11 times higher than the national rate of 1.26% as at 15 April 2020.^2^ The overall positivity rate after total staff screening was 4.4%, over 3 times higher than the national rate at the time. In total, 10 female (71%) and 4 male (29%) staff were affected (Table 2) and this may be as a result of majority of frontline HCW being females.^6^ This contrasted with what was in the general population where 60% of all those infected were males and 40% were females.^2^ Interestingly, neither office staff nor doctor was affected in the department then. A total of 13 (92.9%) out of 14 infected staff were asymptomatic and only 1 person had mild symptoms prior to being screened (Table 2).

**TABLE 2:** Socio-demography and disease presentation of positive staff.

Variable	Category	All *N* = 14 (%)	
		*n*	%
Sex	Male	4	28.6
	Female	10	71.4
Age (years)	< 20	1	7.1
	20–29	3	21.4
	30–39	9	64.3
	40–49	0	0.00
	50–59	1	7.1
Nature of disease manifestation	Asymptomatic	13	92.9
	Symptomatic	1	7.1
**Health worker category**			
Clinical	Nurse	4	28.6
	Pharmacist	1	7.1
	Orderly	3	21.4
	Laboratory scientist	1	7.1
Non-clinical	Banker	2	14.3
	Security officer	1	7.1
	Records officer	1	7.1
	ICT	1	7.1
Associated comorbidities	Hypertension	2	14.3
	Bronchial Asthma	1	7.1
	None	11	78.6

ICT, Information Communication Technology.

Most HCWs are considered at high-risk of getting infected with COVID-19 virus. Their risk of exposure to infection is from both suspected and confirmed cases. The risk is potentially higher at the primary care setting where undifferentiated cases are largely seen. This is further compounded by the fact that appropriate PPE may not be used in attending to asymptomatic COVID-19 cases.

However, it is difficult to ascertain whether all healthcare worker infections are nosocomial in nature. At the time of staff screening, there had been no confirmed cases of COVID-19 infection amongst patients or staff in the department. There was ample evidence at the time that community transmission was ongoing as 71% of those found to be infected in the country had no travel history and 4 out of 5 new cases reported by GHS on 15 April 2020 were asymptomatic.^2^

## Conclusion

It became apparent from our experience that both clinical and non-clinical staff are at risk of getting infected or stand the risk of infecting other categories of staff who may not be in their respective units. A significant step we took, was to modify the prescribed use of PPE for clinical staff. Prior to the detection of COVID-19 infection amongst staff, clinical staff wore disposable gowns, goggles, disposable aprons, head covers, N95 masks or medical masks to attend to suspected cases of COVID-19 only. Upon re-opening the department after our incidental finding, these PPE are being used for both routine and suspected COVID-19 cases. However, the disposable gowns have been replaced by reusable surgical gowns. In addition, hazmat suits, N95 masks, face shields or goggles and boots are used whilst attending to detained suspected cases in the holding bay. The WHO recommends the use of the above combination of PPE for suspected cases.^7^ However, in the primary care setting, with majority of COVID-19 infections being asymptomatic, it may be difficult to predict who should actually be suspected for COVID-19 infection. The use of the reusable surgical gowns has been sustainable as the KPFMD has taken advantage of an existing laundry and Central Sterile Supply Department (CSSD) process already available in KBTH. The cost of a cycle of washing and disinfection of one reusable surgical gown is about GHS 20.00 ($3.40) and is more affordable than the disposable gown, which costs about GHS 120.00 ($20.69) and was discarded after use.

Different guidelines and criteria may need to be developed for screening HCWs during a pandemic, which has a significant number of people as asymptomatic carriers. This will protect HCWs from spreading infections amongst themselves and to vulnerable patients in their care. Delayed turnaround time for COVID-19 test results also slows the needed action of isolation and treatment of affected staff to mitigate further spread.

We conclude that in the primary care setting, HCWs must be ready to see all the following cases safely: routine patients, asymptomatic COVID-19 patients and suspected COVID-19 patients.
